# Adaptations to Hydrothermal Vent Life in *Kiwa tyleri*, a New Species of Yeti Crab from the East Scotia Ridge, Antarctica

**DOI:** 10.1371/journal.pone.0127621

**Published:** 2015-06-24

**Authors:** Sven Thatje, Leigh Marsh, Christopher Nicolai Roterman, Mark N. Mavrogordato, Katrin Linse

**Affiliations:** 1 Ocean and Earth Science, University of Southampton, European Way, Southampton, SO14 3ZH, United Kingdom; 2 National Oceanography Centre, Southampton, European Way, Southampton, SO14 3ZH, United Kingdom; 3 Engineering Sciences, μ-VIS CT Imaging Centre, University of Southampton, Southampton, SO17 1BJ, United Kingdom; 4 British Antarctic Survey, High Cross Madingley Road, CB3 0ET, Cambridge, United Kingdom; Universität Göttingen, GERMANY

## Abstract

Hydrothermal vents in the Southern Ocean are the physiologically most isolated chemosynthetic environments known. Here, we describe *Kiwa tyleri* sp. nov., the first species of yeti crab known from the Southern Ocean. *Kiwa tyleri* belongs to the family Kiwaidae and is the visually dominant macrofauna of two known vent sites situated on the northern and southern segments of the East Scotia Ridge (ESR). The species is known to depend on primary productivity by chemosynthetic bacteria and resides at the warm-eurythermal vent environment for most of its life; its short-range distribution away from vents (few metres) is physiologically constrained by the stable, cold waters of the surrounding Southern Ocean. *Kiwa tyleri*has been shown to present differential life history adaptations in response to this contrasting thermal environment. Morphological adaptations specific to life in warm-eurythermal waters, as found on – or in close proximity of – vent chimneys, are discussed in comparison with adaptations seen in the other two known members of the family (*K*. *hirsuta*, *K*. *puravida*), which show a preference for low temperature chemosynthetic environments.

## Introduction

**Fig 1:**
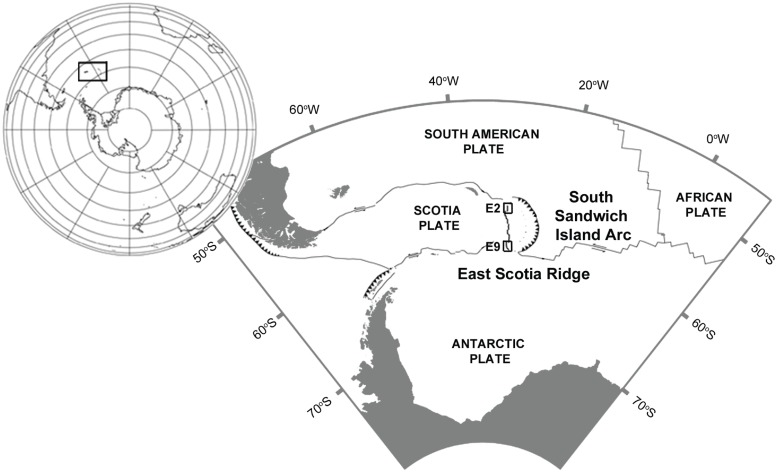
Hydrothermal vents on the East Scotia Ridge (ESR). The Scotia Sea showing the ESR in relation to the Scotia Plate and adjacent plates. Hydrothermal vent sites (E2, E9) as indicated.

The discovery of hydrothermal vent systems on the East Scotia Ridge (ESR) in the Southern Ocean posed new questions on the biogeography and connectivity of vent biogeographic provinces at global scale (Fig 1) [1,2]. In general, vent invertebrate macrofauna are known to thrive within a rather wide thermal range, broadly equivalent of that of invertebrates occurring in shallow waters of temperate regions [3,4]. Situated in the cold polar waters of the Southern Ocean, hydrothermal vents of the ESR are surrounded by permanently low temperatures, around or below 0°C [1,5,6], and therefore comprise the physiologically most isolated chemosynthetic environments known.

Because of these environmental conditions, connectivity of populations inhabiting vent sites in the Southern Ocean may be even more constrained than elsewhere in the oceans [7,8]. Whether or not Southern Ocean vents represent bottlenecks for the distribution and radiation of taxa throughout this polar environment is one of the key questions in the study of global vent biogeography [1].

**Fig 2:**
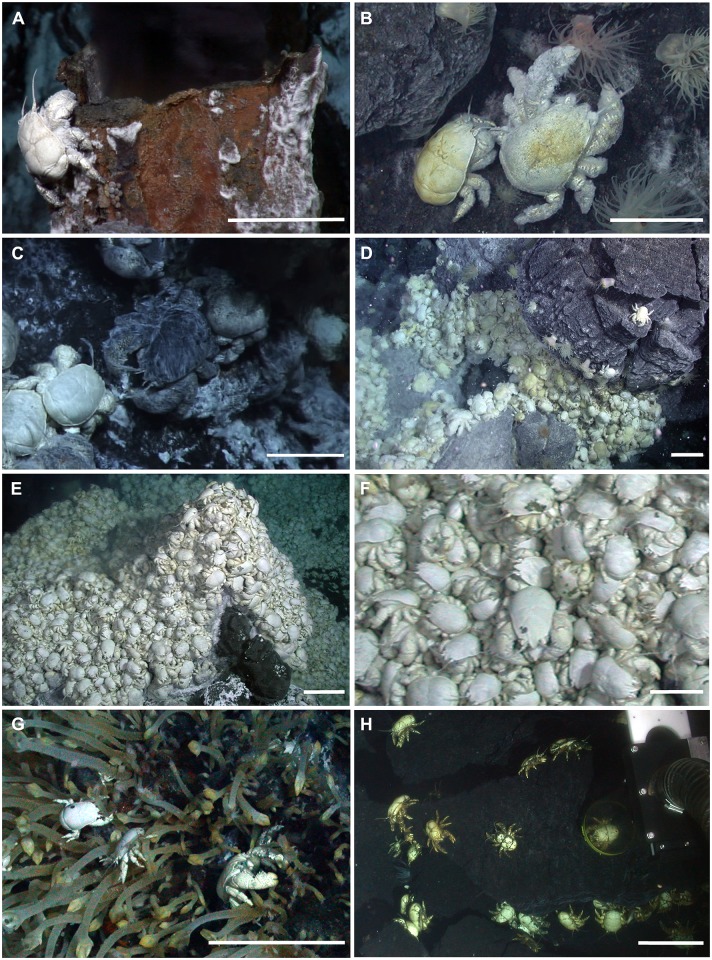
Examples of the differences in assemblages and localities of *Kiwa tyleri* from the Southern Ocean vent fields. A) Individual male *Kiwa tyleri* at the orifice of a "black-smoker" fluid exit on the "Dog's Head" Chimney complex [1,6], E2 vent field; B) Female (left) and male (right) on the lower part of the "Dog's Head" chimney complex; C) "*Kiwa B*" assemblage (left) adjacent to "*Kiwa A*" assemblage (right) at the "Black & White" chimney at the E9 vent field; D) "*Kiwa B*" assemblage "Crab City" at the E2 vent field; E) "*Kiwa B*" assemblage at the "Black & White" chimney at the E9 vent field; F) Zoomed in section to show the dense multilayer aggregations of the "*Kiwa B*" assemblage at the "Black & White" chimney, both male and female individuals can be identified from chela dimorphism; G) Small individual *Kiwa tyleri* associated with the "*barnacle assemblage*" at the "Black & White" chimney; H) Brooding females away from the influence of direct fluid flow at "Anemone Field" E2 vent field; note: ROV slurp gun visible. Scale bar: 5cm (A,B,C,F,G); Scale bar: 10 cm (E,F,H).

An enigmatic squat lobster of the family of yeti crabs, *Kiwa tyleri* sp. nov, visually dominates the two vent systems (Figs 1 and 2) known from the northern and southern segments of the ESR [1,5,6]. The Kiwaidae currently consist of three known species, of which *K*. *tyleri* sp. nov. is the only known representative in the Southern Ocean. *Kiwa puravida* [9] was found at cold seep in the deep sea off Costa Rica, and *K*. *hirsuta* [10] from the periphery of deep-sea hydrothermal vents on the Pacific-Antarctic Ridge [9]. A species of *Kiwa*, visually resembling *K*. *tyleri*, has been reported from the Southwest Indian Ridge, in close proximity to active vents [7,11].

Radiation of the Kiwaidae has been hypothesised as relatively recent, with a suggested divergence between the Pacific and non-Pacific lineages occurring in the Miocene, approximately 13.4–25.0 million years ago (Ma), subsequent to the opening of the Drake Passage and coincident with an important transition in Southern Ocean cooling, resulting in a climate close to as seen today [7,12]. At the two ESR vents (E2 and E9, Fig 1), *Kiwa tyleri* occurs in high densities at the base and lower parts of vent edifices (Fig 2), and can exceed 700 individuals m^-2^. Spatial segregation by size class was observed, and the size of males to females varied significantly between both vent sites [5,6].

The species relies on primary production by chemosynthetic bacteria, and differences in hydrothermal vent fluid composition have been suggested to affect the composition and diversity of epibiotic bacteria at each site [6,13,14]. Previous study suggested that this dependence on chemosynthetic bacteria restricts *K*. *tyleri* to the warm-water environment in closest proximity to vent chimneys, and fewer specimens—mostly brooding females—have been found in short distance away from vents [6]. The early larval development and energetics has been described, suggesting an abbreviated (few stages) and lecithotrophic (food independent) mode of development away from the chemosynthetic and thermally highly variable environment surrounding vent chimneys [8]. Larvae are negatively buoyant—likely of demersal (close to seafloor) living—and, following hatching, are exposed to the low polar temperatures of the Southern Ocean, implying slow development and extended period of larval developmental time [8].

Low polar temperatures are known to pose thermal challenges to most reptant decapod crustaceans (broadly: crabs and lobsters) in the Southern Ocean. Principally, their inability to down regulate the high concentration of magnesium (Mg^2+^) in their blood (haemolymph) below that of sea-water results in a paralysis-like condition in combination with low polar (<1°C) temperatures (for review see [15,16]). This physiological constraint on activity has often been used to explain the low diversity of decapods found in the Southern Ocean [17,18].

So far, only one species of squat lobster of the cosmopolitan genus *Munidopsis* has been reported from the deep-sea of the Bellingshausen Sea, to the west of the Antarctic Peninsula [19]. Whether or not it is the dependence on nutrition provided by chemosynthetic bacteria or the incapability of *K*. *tyleri* to cope with the challenging thermal conditions of the Southern Ocean, or a combination of both, is a paramount question for understanding better how vent invertebrates maintain populations in polar seas. Here, we describe *Kiwa tyleri* sp. nov. and discuss how its morphological specialisations inform our understanding of vent-specific ecological adaptations in a polar environmental setting.

## Materials and Methods

### Ethics statement

This study was undertaken under the permit S3-3/2009 issued by the Foreign and Commonwealth Office, London to section 3 of the Antarctic Act 1994.

### Area under investigation and field sampling

Specimens of *Kiwa tyleri* sp. nov. were obtained from two hydrothermal vent fields situated on the northern and southern branch of the East Scotia Ridge (ESR) during *RRS James Cook* research cruise 42 (7^th^ January- 24^th^ February 2010). The northern vent field is situated on the E2 segment (between 56°05.29' and 56°05.49'S and 30°19.00’ and 30°19.36'W, Fig 1) at ~2600 m depth [1]. The E9 vent field is located at the southern end of the ESR at ~2400 m depth, situated between 60°02.50' and 60°03.00'S and 29°59.00’ and 29°58.60'W (Fig 1). Both vent fields consist of active and inactive hydrothermal structures likely associated with fissures parallel to the ridge axis [1]. Vent chimneys in both fields are characterised by multiple black smokers, with a maximum measured outflow temperature of 380.2°C at E9. Active vent chimneys are characterised by steep changes in temperature, with flanges and lower structures often providing additional exits for emitting diffuse hydrothermal vent fluids at lower temperatures (~3.5 to ~19.9°C [1]). Both sites are surrounded by the cold stable waters of the Southern Ocean, with temperatures of ~0.0 and -1.3°C at E2 and E9 vent sites, respectively [Rogers et al. 2012]; the E9 site is strongly influenced by the Weddell-Scotia Confluence, the Circumpolar Deep Water and the Weddell Sea Deep Water [20–22].

### Morphological analysis

Specimens of *Kiwa tyleri* were collected from six biological sampling dives at both vent fields, using the ROV (remotely operated vehicle) *Isis* equipped with a suction sampler [23,24]. Out of these, type material was selected and fixed in 5% formaldehyde or 99% ethanol, respective of later morphological and molecular analyses in the laboratory. Any morphological measurements were taken to the nearest 0.1mm. Carapace length (CL) was measured from the base of the rostrum to the postero-lateral margin of the carapace. Type material used herein is deposited in the Crustacea collection of the National History Museum, London, United Kingdom (NHMUK) under individual collection codes and station data (see Description).

### Molecular analysis

#### DNA isolation, amplification and sequencing

Genomic DNA from seven individuals from E2 (between 56°05.29' and 56°05.49'S) and E9 (between 30°19.00’ and 30°19.36'W) was isolated from pereopod muscle tissue and DNA was extracted using the Qiagen DNeasy® Blood and Tissue Kit (Cat. 69506) following the manufacturer’s instructions. Three ribosomal gene regions, 16S, 18S, 28S and one protein-coding gene, Cytochrome Oxidase subunit I (COI) were PCR amplified using one or more sets of primers (Table 1). COI, 18S and 28S reactions were performed in 9.5 μl volumes, containing 0.5 μl of each primer (forward and reverse at 10 pmol/μl each), 3 μl of 10X reaction mix, 1 μl of MgCL_2_ (50 pmol/μl), 1 μl dNTPs (10 pmol/μl) 0.25 μl Taq polymerase, 2.25 μl double distilled water and 1 μl of template DNA. The PCR cycling protocol was an initial denaturation at 96°C for 5 minutes, followed by 35 cycles of 96°C for 30 seconds, 55°C for 30 seconds, 72°C for 1 min, and a final extension of 5 min at 72°C. The 16S reaction was performed in a 12 μl volume containing 0.8 μl of each primer (forward and reverse at 4 pmol/μl), 8 μl of Qiagen HotStarTaq Master Mix, 2 μl of DNA template and 0.4 μl of double-distilled water. The PCR cycling protocol was an initial HotStarTaq denaturation at 95°C for 15 minutes, followed by 35 cycles of 94°C for 45 seconds, 55°C for 90 seconds, 72°C for 1 min and a final extension of 7 min at 72°C. All PCR reactions and sequencing reactions were performed on a Bio-Rad C1000 Thermal Cycler. PCR product was purified using QIAquick® PCR Purification Kit (Cat.28106). Where the C1000 Thermal Cycler was used for sequencing reactions, an Applied Biosystems 3100 DNA Analyser was used for sequencing. Forward and reverse sequences were assembled and cleaned using the computer program Sequencher™ 3.0. All sequences are deposited on GenBank, Accession numbers (Table 2).

**Table 1:** List of Primers used in the study.

Gene	Primer	Sequence (5'-3')	Source
Cytochrome Oxidase subunit I	LCO1490	GGTCAACAAATCATAAAGATATTGG	[28]
	HCO2198	TAAACTTCAGGGTGACCAAAAAATCA	[28]
16S rRNA Ribosomal	16S ChiroF	TTCTTGCCTGTTTAACAAAAAC	[7]
	16S ChiroR3	GGTCTGAACTCAAATCATGTAAA	[7]
18S rRNA Ribosomal	18e	CTGGTTGATCCTGCCAGT	[29]
	18S F448	GGAGAGGGAGCCTGAGAAAC	[1]
	18S F896	TTAGAGTGCTCAGAGCAGGC	[1]
	18S F1437	ATGGCCGTTCTTAGTTGGTG	[1]
	18S F1857	TTCCCATGAACGAGGAATTC	[1]
	18P	TAATGATCCTTCCGCAGGTTCACCT	[29]
	18S R498	AAGGGCATCACAGACCTGTT	[1]
	18S R1074	TATCTGATCGCCTTCGAACC	[1]
	18S R1536	ACGAGCTTTTTAACCGCAAC	[1]
28S rRNA Ribosomal	28S-F216	CTGAATTTAAGCATATTAATTAGKGSAGG	[30]
	28S-R443	CCTCACGGTACTTGTTCGCTATCGG	[30]

**Table 2:** List of Sequences and GenBank accession numbers used in phylogenetic analyses.

Species	GenBank Acc #	Reference	GenBank Acc#	Reference
***Kiwa tyleri* sp. nov. 304.2**	**KP763654**	**Present study**	**KP763667**	**Present study**
*Aegla alacufi*	FJ472207	[31]	EU920958	[38]
*Aegla ligulata*	AY595811	[32]	AY595801	[32]
*Agononida procera*	AY351078	[33]	EU821556	[30]
*Alainius crosnieri*	AY351239	[33]	HQ380287	[44]
*Allogalathea elegans*	GU392128	[34]	EU821560	[30]
*Blepharipoda occidentalis*	AF436053	[35]	AF436014	[35]
*Bythiopagurus macrocolus*	EU821532	[30]	EU821548	[30]
*Calcinus obscurus*	AF436058	[35]	AF436022	[35]
*Coenobita perlatus*	HQ241512	[36]	HQ241524	[36]
*Discorsopagurus schmitti*	AF436055	[35]	AF436017	[35]
*Dromia dehaani*	AY583899	[37]	AY583972	[37]
*Emerita analoga*	AF425322	Zaklan & Cunningham, unpublished	AF439383	[39]
*Eumunida sternomaculata*	AY351260	[34]	AF436011	[35]
*Eumunida funabulus*	EU920922	[38]	EU920957	[38]
*Heteronida aspinirostris*	AY351251	[34]	HQ380286	[44]
*Isocheles pilosus*	AF436057	[35]	AF436021	[35]
*Kiwa hirsuta*	EU831286	[30]	EU920942	[38]
*Kiwa puravida*	KF051318	[9]	JN367460	[9]
*Lepidopa californica*	AF436054	[35]	AF436016	[35]
*Lomis hirta*	AF436052	[35]	AF436013	[35]
*Munida acantha*	HQ241515	[36]	HQ241527	[36]
*Munidopsis rostrata*	EU920928	[38]	EU920961	[38]
*Oedignathus inermis*	AF425334	Zaklan & Cunningham, unpublished	Z14062	[45]
*Pachycheles haigae*	AY050076	[39]	AF439389	[39]
*Pagurus longicarpus*	NC003058	[40]	AF436018	[35]
*Petrolisthes armatus*	HM352474	[41]	AF436009	[35]
*Polycheles aculeatus*	AY583885	[37]	AY583959	[35]
*Pseudomunida fragilis*	EU821536	[30]	EU821552	[37]
*Shinkaia crosnieri*	EU420129	[42]	NA	[44]
*Upogebia coralliforma*	EU874906	[43]	EU874956	[43]
*Uroptychus nitidus*	AY595925	[37]	AF439387	[39]
*Uroptychus parvulus*	AY595926	[37]	AF439386	[39]

#### Pairwise distance and phylogenetic analyses

**Fig 3:**
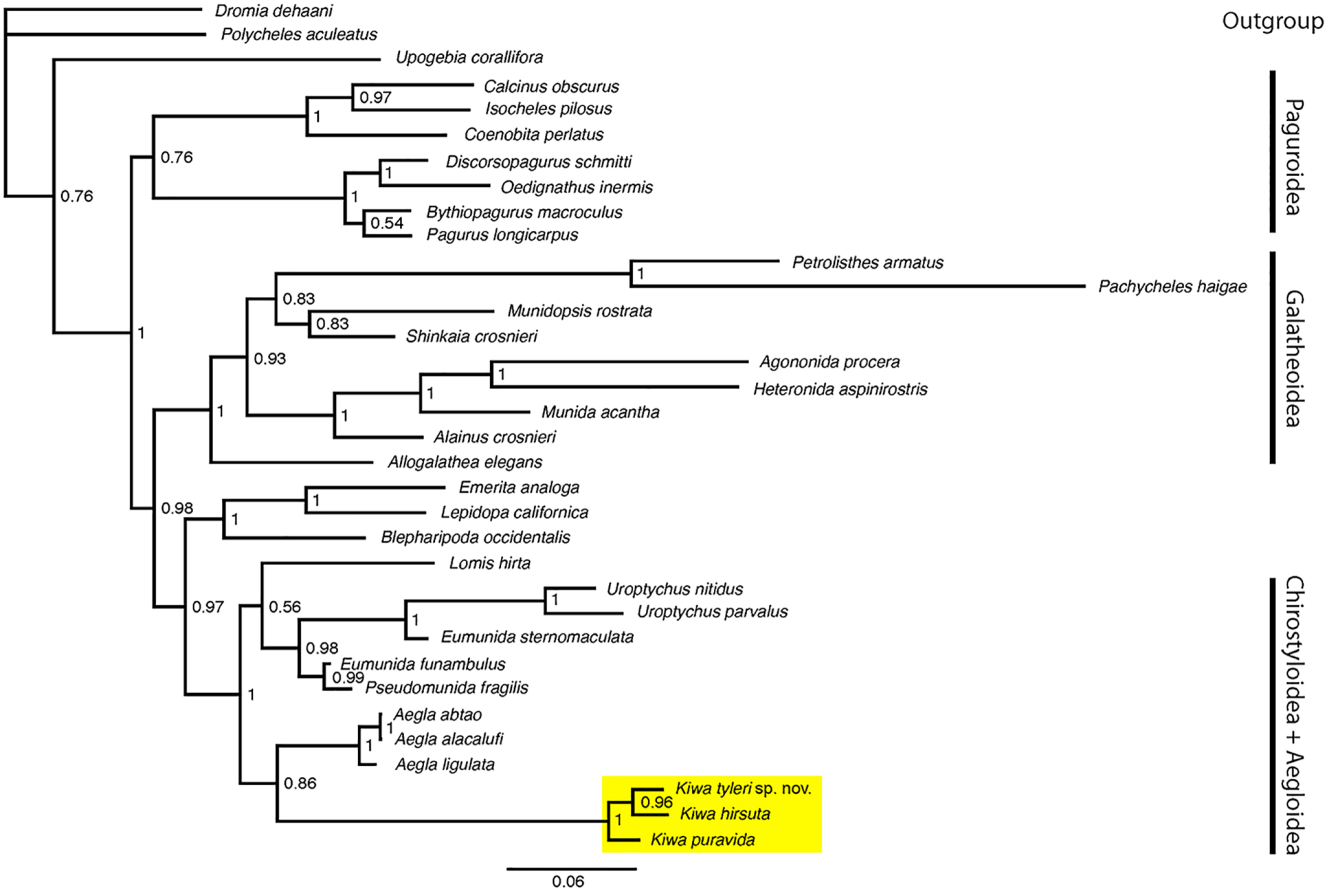
Phylogenetic position of Kiwaidae. Bayesian inference phylogenetic tree based on 16S and 18S fragments. Strong node support (posterior probability of 1.0) is evident for the monophyly of *K*. *hirsuta* and *Kiwa tyleri* sp. nov.

16S and 18S sequences from individual F304.2 from E2 on the East Scotia Ridge were incorporated into an alignment of anomuran crustaceans to construct a phylogenetic tree (Fig 3). Alignments with sequences obtained in this study and sequences from GenBank (Table 2) were constructed using MAFFT 6.861. Bayesian inference of phylogeny was performed using MrBayes 3.2 [24]. The appropriate substitution model for both the 16S and 18S fragments was determined to be GTR (Generalized Time Reversible model) with invariable sites and gamma distribution according to JmodelTest 0.1.1 [25], using the Akaike Information Criterion (AIC). For tree construction, Metropolis coupled Monte Carlo Markov Chains were run for 5 million generations in two simultaneous runs, each with 4 differently heated chains. Topologies were sampled every 100 generations and the first 25% were discarded as ‘burn in’. Pairwise COI Kimura two-parameter (K2P) genetic distances between the seven individuals and also the two presently described kiwaids, *K*. *hirsuta* (COI sequence courtesy of Dr W. Joe Jones) and *K*. *puravida* (Genbank accession #JN383822) were calculated using MEGA 5.0. [9].

### Computed Tomography (CT) Scanning

Holotype (female) (NHMUK 2015.2791) and paratype males (NHMUK 2015.2792; NHMUK 2015.2793) were subjected to micro-focus X-ray Computed Tomography (X-CT) scanning. The scans were performed using a Nikon XT-H 225 L micro focus X-CT system housed within the ‘μ-VIS’ X-ray imaging centre for computed tomography, University of Southampton. The specimens were scanned without removal from their containers or formalin solution. The scans were conducted using a 225kV (peak) X-ray source fitted with a tungsten reflection target, together with a PerkinElmer XRD 1621 CN14 HS detector. Due to the size differential between specimens, slightly different settings were used to scan the large male compared to those used to scan the smaller male and female. For the large male (NHMUK 2015.2792), a peak tube voltage of 80kV together with a current of 300μA and 1mm of aluminium filtration was used with an exposure time of 1 second, 24dB analogue gain, and the specimen positioned to obtain a voxel size of 60μm. For the paratype male (NHMUK 2015.2793) and holotype female (NHMUK 2015.2791) a peak tube voltage of 80kV together with a current of 100μA without filtration was used with an exposure time of 2 seconds, 24dB analogue gain, and the specimen positioned to obtain a voxel size of 44μm. The scans were reconstructed into a 3D volume using a filtered back reconstruction algorithm in CT Pro and CT Agent (Nikon Metrology, UK), and then the volume files were visualized and analysed using VG Studio Max 2.1 (Volume Graphics, GmbH).

The method used allowed for the study of morphological features, without any invasive method applied to type material, and by discounting any setation in the specimens. This approach provides new perspectives for the future studies of Crustacea that are heavily covered in setae, and which may present hidden morphological characters of potential significance in the reconstruction of their evolutionary history, e.g. phylogenetic positioning within clades of species.

### Nomenclature acts

The electronic edition of this article conforms to the requirements of the amended International Code of Zoological Nomenclature, and hence the new names contained herein are available under that Code from the electronic edition of this article. This published work and the nomenclatural acts it contains have been registered in ZooBank, the online registration system for the ICZN. The ZooBank LSIDs (Life Science Identifiers) can be resolved and the associated information viewed through any standard web browser by appending the LSID to the prefix “http://zoobank.org/”. The LSID for this publication is: urn:lsid:zoobank.org:pub:E94A8419-D17A-48A5-A3F1-565DA3E3AB45. The LSID fir the species is: urn:lsid:zoobank.org:act:9D3C4FEC-35B2-4CF9-9EDF-7192A1E88CCB. The electronic edition of this work was published in a journal with an ISSN, and has been archived and is available from the following digital repositories: PubMed Central, LOCKSS.

## Results

### Systematics

Superfamily Galatheoidea Samouelle, 1819

Family KIWAIDAE Macpherson, Jones & Segonzac, 2005

Kiwaidae Macpherson, Jones and Segonzac, 2005: 712

Genus *Kiwa* Macpherson, Jones and Segonzac, 2005: 712

### *Kiwa tyleri* sp. nov. Thatje, 2015

**Fig 4:**
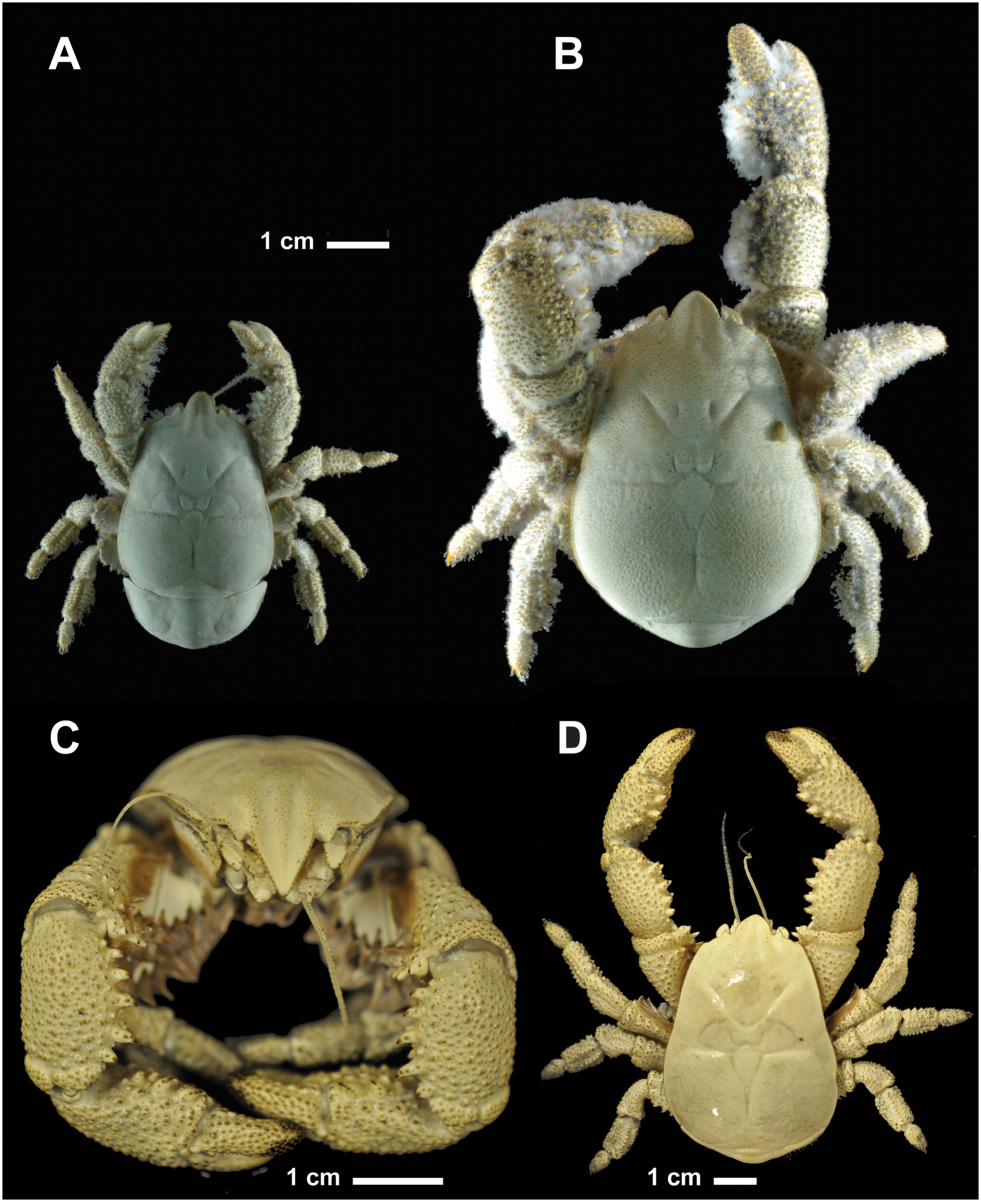
*Kiwa tyleri* sp. nov. A) type material, female, dorsal view; B) type material, male, dorsal view; note presence of single specimen of limpet (*Lepetodrillus* sp.) on carapace; C) paratype, male (NHMUK 2015.2793) frontal view; D) paratype, male (NHMUK 2015.2793) dorsal view.

urn:lsid:zoobank.org:act:9D3C4FEC-35B2-4CF9-9EDF-7192A1E88CCB (Fig 4).

#### Material examined

holotype, female (CL = 4.7 cm) (NHMUK 2015.2791); paratype, male (CL = 4.7 cm) (NHMUK 2015.2792) (30/01/2010; 2395m; station: E9-141; 60°02.80’S, 29°58.70’W); type material, large male (CL = 6.7 cm) (NHMUK 2015.2793) (22/01/2010; 2611m; station: E9-132; 56°05.29’S, 30°19.06’W). Further collection of type material is available (NHMUK 2015.27952804) (30/01/2010; 2395m; station: E9-141; 60°02.80’S, 29°58.70’W). For sampling and locations see methods, and for further information: Table 1 in [6]. Further type material is deposited in museum collections (Table 3).

**Table 3:** List of specimens of *Kiwa tyleri* sp. nov. deposited in museum collections.

Specimen	Collection number	Museum
holotype, female	NHMUK 2015. 2791	NHMUK
paratype, male	NHMUK 2015. 2792	NHMUK
paratype, large male	NHMUK 2015.2793	NHMUK
type material (various)	NHMUK 2015.27952804	NHMUK
type material, male	ZSMA20159501	ZSM
type material, female	ZSMA20159500	ZSM
type material, male/female	SMF 48479	Senckenberg
type material, male	MNHN-IU-2014-10161	NHM Paris
type material, female	MNHN-IU-2014-10162	NHM Paris
type material, male	NIWA99697	NIWA
type material, female	NIWA 99698	NIWA

Abbreviations: NHMUK (National History Museum, London, United Kingdom); ZSM (Bavarian State Collection of Zoology, Germany); Senckenberg (Senckenberg Forschungsinstitut und Naturmuseum, Germany); NHM Paris (Muséum national d'Histoire naturelle, France); NIWA (National Institute of Water and Atmospheric Research, New Zealand).

#### Etymology

*Kiwa tyleri* sp. nov.; the species (*tyleri*) is named after Paul A. Tyler in recognition of his services to higher education and deep-sea biology.

#### Description (Figs 4–8)

**Fig 5:**
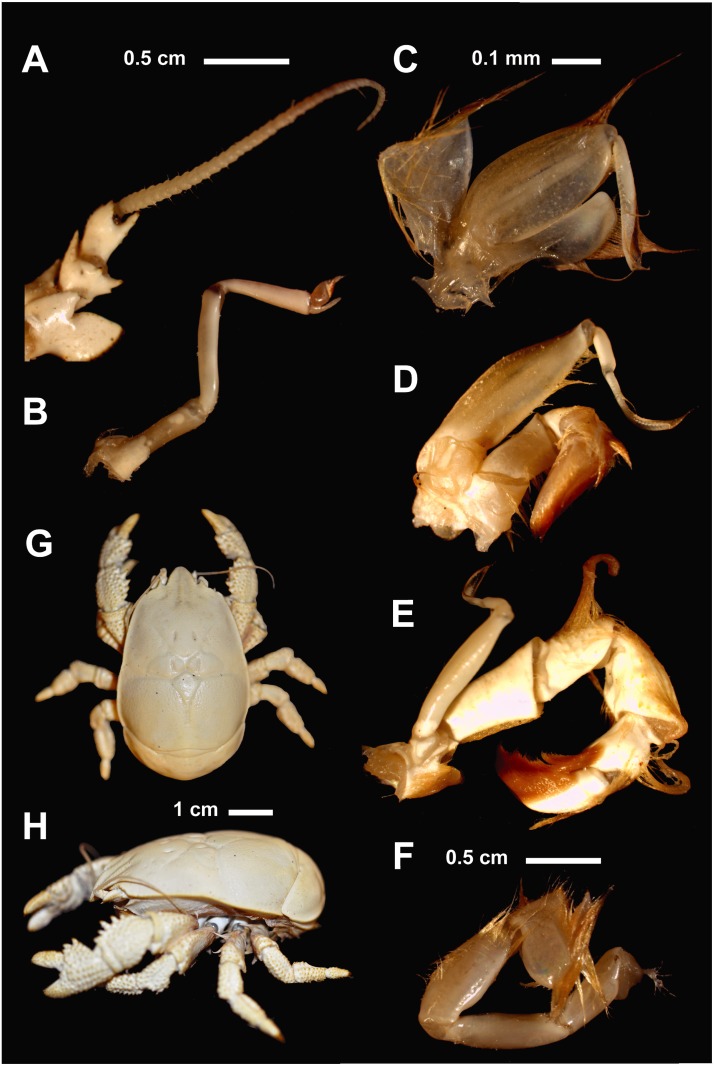
*Kiwa tyleri* sp. nov.; type material, female (A–F). A) antenna; B) antennule; C) first maxilliped; D) second maxilliped; E) third maxilliped; F) pereiopod 5; G) holotype, female (NHMUK 2015.2791); H) lateral view; scale bars: 1mm (A-F); 0.5 cm (G,H).

**Fig 6:**
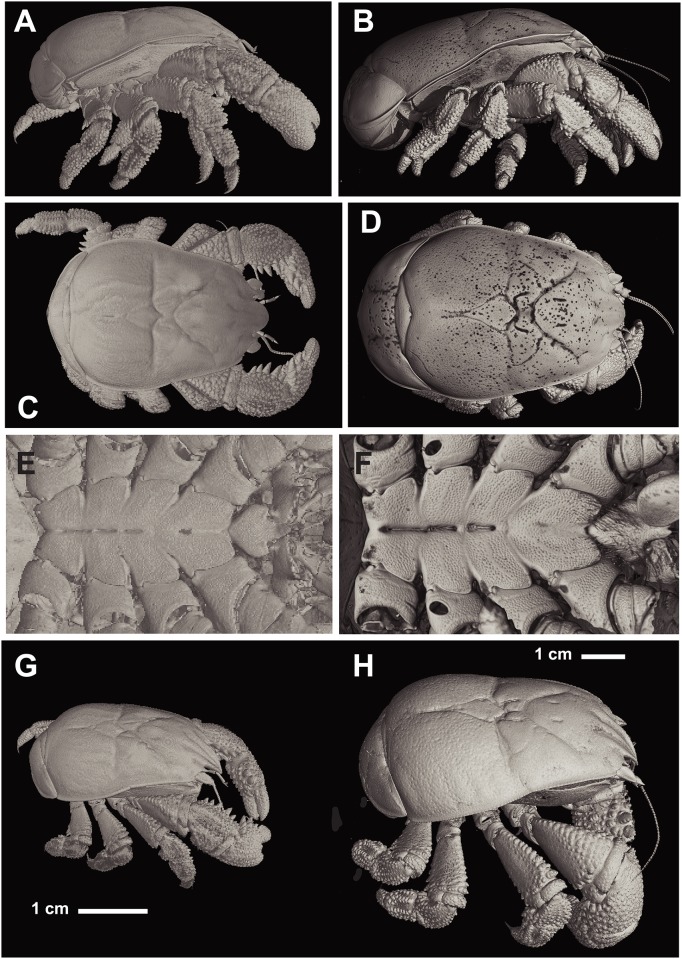
*Kiwa tyleri* sp. nov.; paratype, male (NHMUK 2015.2792). A) lateral view; C) dorsal view; E) ventral view, sternal plastron; G) dorso-lateral view; holotype, female (NHMUK 2015.2791) B) lateral view; D) dorsal view, F) ventral view, sternal plastron, with gonopore on first segments of pereopod 3 (arrow); note: sternal plastron with remnants of setae (methodological artefact, see methods section); H) paratype, large male (NHMUK 2015.2793) (all images based on micro-focus X-CT).

**Fig 7:**
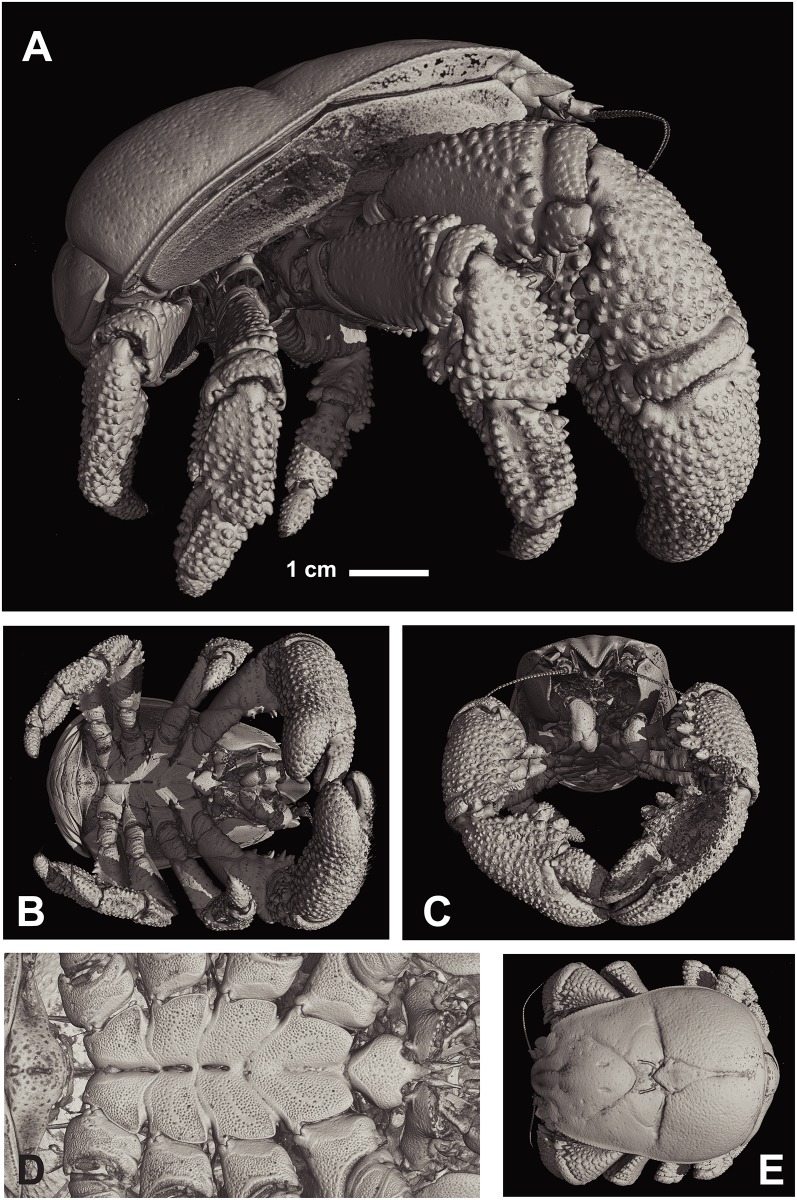
*Kiwa tyleri* sp. nov.; paratype, large male (NHMUK 2015.2793). A) lateral view; B) dorsal view; C) frontal view; D) ventral view, sternal plastron; E) dorsal view.

**Fig 8:**
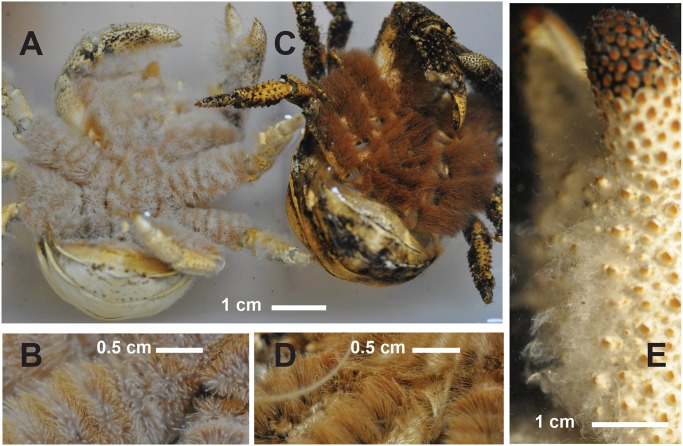
*Kiwa tyleri* sp. nov.; type material. A) female, ventral view; B) female, ventral view, magnification of base of pereopod 4, with rows of distinct (white/brown) setae (black arrows); C) female, ventral view; D) magnification of base of pereopod 4, with worn setae (black arrow); E) type material, male, lateral view of chela, with epibiotic bacteria (black arrow).

Body suppressed, symmetrical, calcified. Eyes absent. Rostrum well developed, pointing ventrally, triangular, slightly narrower than long; with basal pair of spines. Carapace, excluding rostrum, longer than broad; lateral borders granulated; dorsal surface of carapace smooth, without setae. Gastric region depressed, posteriorly separated from anterior branchial and cardiac regions by distinct depression; posterior gastric pit on either side. Cervical groove clearly distinct between gastric and anterior branchial regions; posterior branch of cervical groove between anterior and posterior branchial regions. Cardiac region clearly visible, separated from branchial regions by shallow grooves.

Pterygostomian flap smooth, without setae, slightly excavated directly below mid-length of anterior branchial region, anteriorly produced; two longitudinal carina, between median depression and posterior border (Figs 6A–6D and 7A).

Sternal plastron of four sub-divisions, one for each pair of pereopods 2–4. Sternite 1 between first maxillipeds large, strongly produced anteriorly (straight in females, ventrally inclined in males), slightly concave at base, lateral borders rounded and smooth, posterior margins convergent; sternite 1 slightly longer than wide. Sternites 2–5, with anterior margin produced to tooth, and with lesser tooth on posterior margin; anterior midline grooved; sternite 6 reduced (Figs 6E, 6F and 7D); sternal plastron densely covered with fields of setae (Fig 8A–8D).

Abdominal somites smooth, spineless, and sparsely setose. Somites 2–6 with two transverse carina at each lateral side, directing from anterior and posterior margin; somites 2–5 each with median part delimited by shallow longitudinal groove at each side. Somite 6 with posterior border rounded and produced, with median longitudinal, shallow groove. Uropods well developed; with smooth margins of outer and inner rami fringed with numerous plumose setae; basal segment short, wider than long.

Telson half as long as wide, posterior margin symmetrically bilobed. Numerous plumose setae along lateral and posterior borders. A few uniramous setae scattered on dorsal surface.

Antennule with slender, proximally inflated basal segment, articles 2 and 3 slender, basal segment slightly longer than article 2 and subequal to article 3. Dorsal and ventral flagella short, subequal in length; ventral flagellum segmented, dorsal flagellum with two large basal segments (Fig 5B).

Antenna; peduncle without scaphocerite. Basal article unarmed; article 2 with strong lateral projection, dentate on distal portion, with additional distoventral spine; article 3 with distomesial spine; article 4 with two distomesial spines (dorsal and ventral). Flagellum less than half as long as carapace (Fig 5A).

Mandible with small teeth on anterior edge of incisor process, declining in size and disappearing toward posterior edge; palp 2-segmented.

Maxillule with well developed endopod, with numerous setae; distal endite fringed with simple and plumose setae; proximal endite large with numerous simple setae. Maxilla with slender endopod, with several simple distal setae; distal endite bilobate, proximal lobe larger than distal; proximal endite bilobate; both endites with numerous simple and plumose setae; scaphognathite large and flattened, margins with numerous single and plumose setae.

First maxilliped with large bilobate exopod; distal exopod of two segments, terminal segment elongate; endopod of rounded lobe, fringed with long plumose setae (Fig 5C).

Second maxilliped with exopod slightly longer than endopod; articles of endopod densely covered with simple and plumose setae, dactylus much longer than combined length of propodus and carpus (Fig 5D).

Third maxilliped well developed, with numerous long plumose setae, mostly in ventral and lateral sides of articles; coxa with distal border strongly produced and denticulate, each tooth with corneous margin; basis and ischium fused, triangular, with ventral and lateral margins tuberculate; crista dentata; merus and carpus triangular, similar in length; propodus with numerous plumose setae in distro-ventral border; dactylus moderately depressed, with dense, plumose setae in distal portion (Fig 5E); third maxilliped without comb-row setae [9].

Pereopod 1 (cheliped) in females and males (Figs 4A–4D and 6A–6D), strongly spinose, about as long as carapace (excluding rostrum); about 1.5 times as long in large males (Fig 4B, 4C and 4D). Proximal margins of segments (ischium to carpus) lined with strong spines, enlarged in males, excluding propodus/chela (Fig 4C and 4D); palm broadened distally; dactylus oval in shape, with proximal surface convex (spoon-like), with apparent smooth cutting edge (Fig 6C).

Pereopods 2–4 (Figs 4A, 4B, 6A–6C, 6G and 6H); ambulatory legs similar; of stout segments and oval in shape. Surface covered with tubercular processes on ischium, merus, carpus, and propodus. with spines and large granules; ventral margin with row of tubercular processes. Carpus with dorsal margin serrated with spines and tubercular processes. Propodus with strong terminal spine (Fig 6A). Pereopod 5, reduced, chelate, inserted below sternite 6, base not visible ventrally; hand and fixed finger strongly reduced, and flattened, longer than broad; numerous and dense setae on extensor margins of palm and movable finger (Fig 4F).

On pereopod 1 to 4; dense fields of setae distributed along ventral portions, from ischium to merus (Fig 8A–8D). Paired pleopods present.

#### Setae

Two distinct types of setae have previously been detected (see Fig 1C and 1D in Zwirglmaier et al. [14]), which are confirmed by the present study; comb-row setae as found in *K*. *hirsuta* and *K*. *puravida* are absent [9,10]. Dense fields of setae are distributed on the sternal plastron, and along ventral portions of pereopods 1 to 4, from ischium to carpus (Fig 8A–8E). These fields consist of two types of setae aligned in alternation, with rows of thicker bristles, surrounded by rows of thinner and more flexible plumose setae (bacteriophoran setae). Epibiotic bacteria are found associated with these setae (Fig 8A and 8B). Brooding females found away from vent chimneys often present a deteriorate state in carapace health [6], including highly worn (brown) setae (Fig 8C and 8D).

#### Species identification

*Kiwa tyleri* sp. nov. can easily be distinguished from the other two known members of the Kiwaidae, *K*. *hirsuta* Macpherson, Jones & Segonzac, 2005, and *K*. *puravida* Thurber, Jones & Schnabel, 2011 [9,10], by i) a smooth (no dorsal spines on surface) carapace (“scarabid-like” form), which is proportionally wider (“bulkier”) than in congeners, ii) pereopod 1 (chela) robust and large; enlarged (more pronounced) in bigger males, iii) pereopds 2–4 shorter, of shorter stouter segments, with propodus ending in strong distal spine, iv) ventral side (sternal plastron) with dense fields of setae consisting of two types of setae aligned in alternation, extending along ventral side of pereopods 2–4 (Figs 4 and 8). *Kiwa tyleri* sp. nov. does not present any comb-row setae on third maxilliped previously used to characterize *K*. *hirsuta* and *K*. *puravida* [9,10].

### Genetic comparison of Kiwaidae

PCR and sequencing yielded a 512 bp fragment of 16S, a 598 bp fragment of 18S, a 1088 bp fragment of 28S and a 680 bp fragment of COI for individuals from both vent locations on the East Scotia Ridge (E2 and E9). The Bayesian inference phylogenetic tree (Fig 3) using concatenated 16S and 18S fragments gave strong node support (posterior probability of 1.0) for the monophyly of *K*. *hirsuta* and *Kiwa tyleri* sp. nov. from E2 on the East Scotia Ridge (individual F304.2). Pairwise K2P genetic distance comparisons of a 412 bp fragment of COI revealed the seven East Scotia Ridge individuals to be between 0–1% divergent from each other, 9.6–10.8% divergent from *K*. *hirsuta* and 11.8–13% divergent from *K*. *puravida* (Table 4).

**Table 4:** COI pairwise Kimura two-parameter distances between seven *Kiwa tyleri* sp. nov. individuals collected from the E2 and E9 vent fields on the East Scotia Ridge (ESR), as well two described Pacific species of *Kiwa*: *Kiwa puravida* and *Kiwa hirsuta*.

	*K*. *puravida*	*K*. *hirsuta*	F22.2	F304.1	F304.2	F304.3	F423.1	F423.2	F423.3
***Kiwa puravida*** JN383822	-								
***Kiwa hirsuta***	0.142	-							
***Kiwa tyleri* sp. nov.** F22.2 (ESR, E2)	0.118	0.096	-						
***Kiwa tyleri* sp. nov.** F304.1 (ESR, E2)	0.121	0.099	*0*.*002*	-					
***Kiwa tyleri* sp. nov.** F304.2 (ESR, E2)	0.124	0.102	*0*.*005*	*0*.*002*	-				
***Kiwa tyleri* sp. nov.** F304.3 (ESR, E2)	0.124	0.108	*0*.*010*	*0*.*007*	*0*.*005*	-			
***Kiwa tyleri* sp. nov.** F423.1 (ESR, E9)	0.124	0.105	*0*.*007*	*0*.*005*	*0*.*002*	*0*.*002*	-		
***Kiwa tyleri* sp. nov.** F423.2 (ESR, E9)	0.130	0.099	*0*.*010*	*0*.*007*	*0*.*005*	*0*.*010*	*0*.*007*	-	
***Kiwa tyleri* sp. nov.** F423.3 (ESR, E9)	0.130	0.099	*0*.*010*	*0*.*007*	*0*.*005*	*0*.*010*	*0*.*007*	*0*.*000*	-

Italicised text highlights pairwise comparisons amongst the ESR individuals.

#### Distribution

*Kiwa tyleri* sp. nov. occurs in high abundance of up to 700m^-2^ at two hydrothermal vent fields situated on the northern and southern branch of the East Scotia Ridge (E2 vent site: between 56°05.29' and 56°05.49'S and 30°19.00’ and 30°19.36'W at ~2600 m depth; E9 vent site: between 60°02.50' and 60°03.00'S and 29°59.00 and 29°58.60'W at ~2400m (Figs 1 and 2) [1,23]. The ecology and life history of this species can be regarded as well reported [1,5,6,9,13,14].

## Discussion

### Phylogenetic position of *Kiwa tyleri* sp. nov.

The monophyly of *Kiwa hirsuta* and *Kiwa tyleri* sp. nov. produced in our phylogenetic analysis is consistent with the presented morphological inferences (see species identification) that the species described herein is a member of the anomuran squat lobster family Kiwaidae (Fig 3) [10]. For a more detailed examination of the evolutionary relationships amongst members of this family, we refer to detailed phylogenetic analysis presented by Roterman et al. [7]. The K2P COI distance matrix revealing 0–1% divergences amongst the seven ESR kiwaids analysed, and distances of 9.6–13% between these individuals and the other two described kiwaid species (*K*. *hirsuta*, *K*. *puravida*) [9,10] are consistent with intra and interspecific diversity respectively within Decapoda [26]. This molecular evidence supports an inclusion of *Kiwa tyleri* within the genus *Kiwa*. There did not appear to be any pattern of differentiation between individuals between vents from the northern (E2) and southern (E9) branch of the ESR (Fig 1); some individuals between sites being as little as 0.2% divergent, yet being 1% divergent from other individuals at the same site (Table 1), consistent with E2 and E9 individuals comprising one species.

### Functional morphology, nutrition, and ecological implications

The three known species of Kiwaidae, including *K*. *tyleri* described herein, are of deep-sea occurrence (1000 to 2400 m), and are clearly associated with chemosynthetic environments. *Kiwa puravida* and *K*. *hirsuta* occur in low-density aggregations of few specimens (<10) per square metre, whereas *K*. *tyleri* presents densely packed aggregations exceeding 700 individuals m^-2^, with a maximum observed abundance of 4017 individuals m^-2^ [6,9,10].

Dependence on chemosynthetic processes has been suggested for all three species, with *K*. *puravida* found at cold seep on the Costa Rica margin, farming methane and sulfide binding ε- and γ-proteobacteria closely related to decapod epibionts at hydrothermal vents [9,27]. *Kiwa hirsuta*, on the other hand, is found toward the periphery of hydrothermal vent fields in the South-East Pacific Ocean, and has been suggested to harbour sulphur-oxidizing bacteria on bacteriophoran setae [10]. *Kiwa tyleri* sp. nov. also relies on primary production by chemosynthetic bacteria, and a variable and diverse array of epibiotic bacteria has been reported for different chimney systems [13,14].

Thurber et al. [9] proposed that seeps could serve as dispersal stepping-stones between vents for kiwaids, and Roterman et al. [7] noted that a seep-to-vent evolutionary pathway for the family was consistent with the basal split between the seep-inhabiting *K*. *puravida* and the remaining kiwaids. Indeed, this hypothesised evolutionary transition may be reflected in changes in the distribution and density of bacteriophoran (plumose) setae, indicating a possible adaptive trend in functional morphology towards greater specialisation for vent-endemic life. Whereas *K*. *puravida* predominantly possesses bacteriophoran setae on its chelipeds, setation in *K*. *hirsuta* extends to all pereopods, and the sternal plastrum, although at a low density. *Kiwa tyleri* sp. nov. has specialised setae in the same areas as *K*. *hirsuta*, but at higher densities; the two types of setae found in this species and their alignment on the ventral side of the body—with rows of thicker bristles, surrounded by rows of thinner and more flexible plumose (bacteriophoran) setae—suggest a highly specialized way of life at Southern Ocean vent chimneys. E2 and E9 vent sites are widely covered in mats of bacteria (Fig 2D, see also [5,6]), and specialization in setae types may suggest that *K*. *tyleri* can harbour/farm epibiotic bacteria, as well as swipe-up bacteria from chimney surfaces. Constrained by the low, stable temperatures prevailing in the Southern Ocean, *K*. *tyleri* is limited to a three-dimensional thermal envelope surrounding vent chimneys (for discussion see [6]). Competition for ‘bacteria’ may be severe where high densities of crabs cover this area in dense layers; at these sites (base of chimney, Fig 2E and 2F), farming of chemosynthetic bacteria is likely the only option and must match energetic demands. Interestingly, few but larger males occupy a warmer thermal environment at the mid and upper parts of the chimney (Fig 2) and may benefit from excess in bacteria found there (for images see [5,6,14]).

In contrast, the other two members of the family have been suggested to farm epibiotic bacteria primarily on their chelipeds, which is particularly evident in *K*. *puravida* possessing both morphological and behavioural adaptations (“cheliped waving”) to harvest its epibionts [9]. Whether the farming of bacteria on chelipeds/pereopods is sufficient for sustenance, or whether supplementary food derived from other sources may also be required, remains to be investigated. It has to be noted however, that *K*. *hirsuta* has been observed to consume bathymodioline mussels that had been damaged by the Alvin submersible, indicative of some degree of omnivory [10].

Another important ecological adaptation to life on vent chimneys is the presence of a well developed spine on the propodus of pereopods 2 to 4, combined with an overall much stouter and compact body form, including robust and proportionally shorter pereopods (2 to 4). This allows *K*. *tyleri* to thrive on the steep surfaces of vent chimneys, maximising their limited thermal habitat available at Southern Ocean vents [5,8]. The other two species of Kiwaidae present a body form typical of squat lobsters living in less challenging geological environment, such as abyssal plains (soft and plain sediments), represented by long, slender pereopods 2 to 4, and a long, slender cheliped that would otherwise affect any climbing on steep surfaces. Segments in pereopods of *K*. *tyleri* are of oval shape, whereas congeners show laterally flattened segments; again, a likely adaptation to contrasting small-scale habitat topography.

A recent study confirmed the presence of *Kiwa* cf *tyleri* on the Southwest Indian Ridge [7,11] and in close proximity of active vents, but at lower densities than at ESR vent sites, and within a slightly warmer deep-sea environmental setting (for discussion of thermal tolerances among anomurans see [16]). Further comparative ecological and physiological study of the Kiwaidae may present important clues on the evolutionary steps taken in colonizing hydrothermal vents.
